# Supplementary material for: Active site specificity profiling datasets of matrix metalloproteinases (MMPs) 1, 2, 3, 7, 8, 9, 12, 13 and 14
Source: Data Brief. 2016 Feb 22;7:299–310. doi: 10.1016/j.dib.2016.02.036 (PMC4777984; doi:10.1016/j.dib.2016.02.036)
Supplement: Supplementary file 10 — Supplementary material [file mmc10.zip › WebPICS_hMMP3_G_1%/P3prime.html]

 

PICS results


|  |  |
| --- | --- |
| **P3prime\_A**  26 in 151 sites   17.2 %    effects > 10 perc. pnts.   (vice-versa in brackets)  P3\_I: 10.6 (21.3)   P3\_L: 19.5 (29.9)   P3\_P: -17.0 (-10.2)   P2\_G: 10.6 (21.3)   P1\_N: 22.6 (24.5)   P1prime\_I: 13.6 (13.6)   P2prime\_H: 12.1 (62.8)   P2prime\_T: 12.6 (32.8) |  |
| **P3prime\_G**  11 in 151 sites   7.3 %    effects > 10 perc. pnts.   (vice-versa in brackets)  P1\_A: 17.4 (12.7)   P1\_K: 39.5 (48.3)   P1prime\_I: 28.3 (11.9)   P1prime\_Q: 11.6 (12.7)   P2prime\_L: 23.8 (13.8) |  |
| **P3prime\_I**  12 in 151 sites   7.9 %    effects > 10 perc. pnts.   (vice-versa in brackets)  P2\_F: 12.7 (25.4)   P1prime\_Q: 10.1 (12.1)   P2prime\_T: 10.1 (12.1)   P2prime\_V: 28.5 (17.1) |  |
| **P3prime\_K**  25 in 151 sites   16.6 %    effects > 10 perc. pnts.   (vice-versa in brackets)  P1\_N: -11.9 (-12.4)   P2prime\_L: 11.4 (15.0) |  |
| **P3prime\_L**  17 in 151 sites   11.3 %    effects > 10 perc. pnts.   (vice-versa in brackets)  P2\_L: 12.2 (12.2)   P1prime\_C: 16.9 (28.7)   P2prime\_Q: 16.9 (28.7) |  |
| **P3prime\_M**  5 in 151 sites   3.3 %    effects > 10 perc. pnts.   (vice-versa in brackets)  P1\_C: 18.0 (30.0) |  |
| **P3prime\_N**  11 in 151 sites   7.3 %    effects > 10 perc. pnts.   (vice-versa in brackets)  P2\_F: 23.3 (42.7)   P1\_D: 17.4 (12.7)   P1\_F: 14.2 (26.0)   P2prime\_I: 42.6 (26.0) |  |
